# Supplementary material for: Effect of the trajectory of exertional breathlessness on symptom recall and anticipation: A randomized controlled trial
Source: PLoS One. 2020 Sep 11;15(9):e0238937. doi: 10.1371/journal.pone.0238937 (PMC7486077; doi:10.1371/journal.pone.0238937)
Supplement: S2 File — (DOCX) [file pone.0238937.s002.docx]

**Recalled exertional breathlessness and improved predicted exercise capacity**

**A randomized controlled trial**

**Swedish title: Andfåddhet under ansträngning**

**Protocol version 1.1 (Ethics approved 2017-05-30)**

| **Background** | Breathlessness during exertion is a major limiting factor for patients’ physical capacity and activity.[[1](#_ENREF_1)] Increased exertional breathlessness often results in impaired activity, spiraling deconditioning and further worsening of exertional breathlessness, quality of life and prognosis.  Recalled symptoms (from memory) can differ substantially from the actually experienced symptoms.[[2](#_ENREF_2), [3](#_ENREF_3)]The recall of symptom intensity is affected by several factors including the experienced peak intensity and the intensity at the end of the episode.[[2](#_ENREF_2), [3](#_ENREF_3)] This ‘Peak-end-rule’ has been reported in studies of pain,[[2](#_ENREF_2), [3](#_ENREF_3)] and in breathlessness in daily life [[4](#_ENREF_4)] and during exercise.[[5](#_ENREF_5)] A randomized trial reported that adding a time period with decreased pain at the end of a colonoscopy decreased the patient’s recalled total pain of the event, improved their overall perception of the event and made them more willing to participate in similar procedures in the future.[[2](#_ENREF_2)]  Pulmonary rehabilitation training is the first line treatment for exertional breathlessness and deconditioning in cardiorespiratory disease.[[6](#_ENREF_6)] However, the training as well as physical activities of daily life are often limited by the person’s perception of his/her capacity, which is based on recalled breathlessness during exertion. The person’s predicted breathlessness – that is, the level of breathlessness that the person predicts will occur during a future activity, is likely a major determinant of his/her willingness to participate in training as well as of the level of physical activity in daily life.  To improve the effectiveness of cardio-pulmonary rehabilitation training and the patients’ health status, new approaches for decreasing the perceived exertional breathlessness and optimize training are needed.[[6](#_ENREF_6)]  We hypothesize that adding a period of lower breathlessness intensity at the end of training might be a way to manipulate the recalled (remembered) symptom intensity during the training, and to improve the subject’s perceived future exercise capacity and willingness to participate in physical exercise/ training. |
| --- | --- |
| **Purposes** | **Primary purpose:** To test whether adding a period of lower level of exertional breathlessness at the end of an exercise test decreases the overall level of recalled breathlessness for the test.  **Secondary purposes:** To evaluate factors that affect the level of recalled breathlessness, and whether decreased exertional breathlessness results in the participant’s reporting lower predicted future exertional breathlessness and higher exercise capacity. |

| **Questions** | **Primary:** After completion of the regular exercise test, does adding a time period of lower breathlessness intensity decrease the recalled overall symptom intensity during the exercise test?  **Secondary:**   - Is the intervention associated with higher predicted future exercise capacity in relation to a standardized exercise scenario? - Is the intervention associated with decreased predicted exertional breathlessness in relation to a standardized exercise such as climbing a number of stairs / cycle uphill? - Which factors predict the level of recalled breathlessness intensity? - Which factors predict the difference score (bias) between recalled and experienced breathlessness intensity? - How does exertional breathlessness predict clinical outcomes: diagnosed cardiovascular disease; risk of cardiovascular intervention, hospitalization, and earlier death? - How much does the breathlessness contribute to the perceived level of exertion during the exercise test? |
| --- | --- |
| **Study design** | Randomized controlled trial with longitudinal follow-up study |
| **Setting** | Standard exercise testing in the clinical physiology laboratory |
| **Eligibility** | **Inclusion criteria:**   - Persons referred and eligible for standard exercise stress testing - Able to read, write and understand sufficient Swedish to participate   **Exclusion criteria for randomization:**   - Duration of exercise test ≤ 3 minutes - Maximal breathlessness intensity ≤ 3/10 during the test - Clinical or cardiovascular instability during the exercise test before randomization as judged by the investigator |
| **Intervention** | After completion of the standard exercise test, an intervention period of 2 minutes of low intensity exercise is added. |
| **Comparison** | No added exercise period (normal exercise test). |
| **Outcomes** | **Primary:**   - Recalled overall intensity of breathlessness during the exercise test   **Secondary:**   - Predicted future exertional breathlessness - Predicted future exercise capacity - Recalled peak breathlessness intensity during the exercise test - Factors influencing the difference between experienced and recalled breathlessness of the exercise test - Recalled descriptors of breathlessness and their intensity - Recalled overall and peak level of exertion during the exercise test - Overall perception of the exercise test - Diagnoses (IHD; heart failure; pulmonary disease; other) during 10 years follow-up (National Patient Register) - Rate of revascularization and coronary bypass operation during 10 years follow-up - Rate of hospitalization (overall and as for diagnosis) during 10 years follow-up (National Patient Register) - Rate of mortality and causes of death (overall and as for diagnosis) during 10 years follow-up (Causes of Death Register) |
| **Study procedures** | **Trial registration**  The trial is registered with ClinicalTrials.gov before recruitment of the first participant.  **Eligibility and consent**  Eligibility criteria, information about the study and the pre-test questionnaire will be sent home to patients referred for standard cycle exercise testing. The pre-test questionnaire is completed by the participant at home or before the exercise test. Eligibility is confirmed and written informed consent (Appendix 1) is obtained for all participants by the investigator before starting the exercise test. All patients included in the RCT, randomizations, and patients included in the observational sub-study are registered in a log file including study ID, Swedish identification number and test date, stored securely at the Department of Clinical Physiology.  **The exercise test**  Standard exercise test is performed according to clinical practice, current international guidelines [[7](#_ENREF_7), [8](#_ENREF_8)] and guidelines from the Swedish association for Clinical Physiology.[[9](#_ENREF_9)] Testing is done on bicycle ergometers with small incremental increases in workload every minute. The level of breathlessness is self-reported by the participant each 2 minutes on a modified Borg CR10 (mBorg) scale between 0 (none) and 10 (maximal).[[10](#_ENREF_10), [11](#_ENREF_11)] The level of perceived exertion is self-reported by the participant on the Borg RPE scale between 7 (none) and 20 (maximal), and standard parameters of exercise testing are measured according to clinical routine practice.[[10](#_ENREF_10)]  **Randomization and intervention**  At the end of the regular exercise test conducted according to clinical practice, with the participant still on the test cycle, a sealed opaque envelope is broken by the staff with a code that randomly allocates the participant in a 1:1 ratio to either an additional 2 minutes of low intensity exercise testing (intervention group), or to no additional testing (control group). During the intervention period. The workload used will be about 50% of the maximal workload but lowered if needed, and assessments are conducted including of breathlessness, perceived exertion, breathing frequency and blood pressure. A period of a few minutes of lower exertion (‘cool down’) at the end of the exercise test is clinical practice in many laboratories including in the USA, and is not expected to affect the safety or diagnostic properties of the exercise test. As an added precaution we have obtained opinions from well-known experts in cardiac stress testing (Dr. Olle Pahlm and Dr. Paul Kligfield). In additions to this we will also use ST loops to further enhance the diagnostic properties of the test. ST loops are not currently used by default in our laboratory.  **Recall of symptoms**  A post-test questionnaire is completed by the participant 30-90 min after the exercise test (Appendix 2).  **Database management**  Data from the questionnaires, the exercise test, intervention and medical records are linked using each participant’s Swedish identification number. The study database is de-identified with a participant-specific study ID replacing the Swedish identification number, stored securely on a password protected computer without internet access at the department of the investigator. The key between each patient’s study ID and Swedish identification number is stored in a folder in a locked storage room for research at the clinic. Analyses are performed by members of the research team (listed below). |
| **Observational sub-study** | Patients completing the exercise test who fulfill the inclusion criteria and give their written informed consent (Appendix 1) are included in an observational longitudinal follow-up study. The study data are cross-linked with medical records and governmental public registries regarding diagnoses, procedures and hospitalizations (National Patient Register) and mortality (Causes of Death Register) with up to 10 years follow-up. |
| **Assessments** | *Pre-test Questionnaire:*   - Self-reported medical diagnoses - Smoking status and pack-years of smoking - The presence of breathlessness experienced during daily life anytime during the last 2 weeks (yes/no) - mMRC breathlessness scale [[12](#_ENREF_12)] - Predicted overall intensity of breathlessness (0-10 mBorg) during the exercise test   *During the exercise test (which includes the intervention period):*   - Height and weight before start of the test - Participant-reported ratings of breathlessness (0-10 mBorg scale) at start, every 2 minutes and at completion of the exercise test - Standard measurements:   - Level of stress (work rate)   - Perceived exertion (7-20 Borg scale) every 2 minutes and at completion of the exercise test   - Respiratory rate every 2 minutes   - Oxygen saturation (SaO_2_) as indicated   - Blood pressure every two minutes   - Electrocardiography (ECG) at baseline (just before the test) and continuously throughout the test (including pulse rate)   - Participant-reported level of overall exertion on a 7-20 Borg RPE scale every 2 minutes and at peak exercise or the end of the intervention period - Adverse events are logged according to clinical routine - The timing of each measurement and event is measured relative to the start of the exercise test - Main reason for stopping the test (breathlessness; leg fatigue; other [free text]) after completing the test - If discontinuing, reason for non-completion (free text)   *Randomization:*   - Reason for non-randomization (free text) - Allocated study group   *Post-test Questionnaire (Appendix 2):*   - Recalled overall intensity of breathlessness (0-10 mBorg scale) and exertion (7-20 Borg scale) during the exercise test - Perception of overall performance during the exercise test (4 point Likert-scale: very bad, slightly bad, slightly good, very good) - Predicted future breathlessness when walking up two flights of stairs after a few days (0-10 mBorg) - Predicted future breathlessness when performing a similar exercise test after a few days (0-10 mBorg) - Predicted future level of exhaustion when walking up two flights of stairs after a few days (0-10 mBorg) - Predicted future exercise capacity (0-10 numerical rating scale; 0=”worst possible exercise capacity” and 10=”best possible or imaginable exercise capacity”) - Unpleasantness, descriptors and emotional responses of the breathlessness during the exercise test (volunteered descriptors;[[13](#_ENREF_13)] and the Multidimensional Dyspnea Profile; MDP])[[14](#_ENREF_14)] - Personality trait of symptom sensitivity (PHQ-15)[[15](#_ENREF_15)]   *Medical records:*   - Reason for performing the exercise test - Diagnoses before the test - Medications before the test - Latest spirometry values - Latest cardiac ultrasound including measured ejection fraction (EF)   *Medical records and National Registry Data (10 years follow-up):*   - Diagnoses, procedure codes and hospitalizations (National Patient Register) - Date and causes of death (Causes of Death Register) |
| **Statistical analyses** | Baseline characteristics will be presented using standard descriptive statistics.  The primary endpoint is the difference in recalled overall breathlessness between groups, which will be analyzed using Student’s t-test.  Factors influencing the continuous primary secondary endpoints will be analyzed using correlational analysis, t-tests and multivariate multilevel linear regression accounting for repeated measurements.  Categorical outcomes (such as the Overall perception of the exercise test) will be analyzed using multivariate multilevel logistic regression accounting for repeated measurements.  Time-to-event outcomes (diagnoses, procedures, hospitalization and death) during follow-up will be analyzed using multivariate Cox regression and Fine-Gray regression accounting for competing events.  The primary analysis will be of the intention-to-treat population, i.e. of all randomized participants. Secondary analysis will be of participants completing the intervention period. |
| **Power and sample size** | A sample size of 74 randomized participants will be required for 80% power to detect a difference of 1 NRS point of recalled overall breathlessness between groups, assuming a significance level (alpha) of 0.05 and a standard deviation of 1.5, based on Stulbarg *et al* [[5](#_ENREF_5)] and Meek *et al*.[[4](#_ENREF_4)] |
| **Recruitment period** | Based on approximately 10 exercise tests performed at the Department of Clinical Physiology each day, a conservative estimate of the expected recruitment period for the RCT is 2-3 months. |
| **Research Team** | Magnus Ekström, primary investigator, MD, PhD, Department of Medicine, Blekinge Hospital, Karlskrona; [pmekstrom@gmail.com](mailto:pmekstrom@gmail.com)  Viktor Elmberg, MD, Department of Clinical Physiology, Blekinge Hospital, Karlskrona; [viktore@gmail.com](mailto:viktore@gmail.com) |
| **Planned publications** | 1. Main paper  2. Secondary outcomes  3. Observational study  4. Long-term cohort follow-up  Planned first author: Viktor Elmberg  Planned last author: Magnus Ekström  Planned co-authors: members of the research team.  Authorship will finally be determined in accordance with the International Committee of Medical Journal Editors (ICMJE) guidelines (<http://www.icmje.org/recommendations/>). |

**REFERENCES**

1. Parshall MB, Schwartzstein RM, Adams L, Banzett RB, Manning HL, Bourbeau J, Calverley PM, Gift AG, Harver A, Lareau SC, Mahler DA, Meek PM, O'Donnell DE, American Thoracic Society Committee on D. An official American Thoracic Society statement: update on the mechanisms, assessment, and management of dyspnea. *American journal of respiratory and critical care medicine* 2012: 185(4): 435-452.

2. Redelmeier DA, Katz J, Kahneman D. Memories of colonoscopy: a randomized trial. *Pain* 2003: 104(1-2): 187-194.

3. Kahneman D. Experienced Utility and Objective Happiness: A Moment-Based Approach. In: D. Kahneman and A. Tversky (Eds.) Choices, Values and Frames. New York: Cambridge University Press and the Russell Sage Foundation 2000.

4. Meek PM, Lareau SC, Anderson D. Memory for symptoms in COPD patients: how accurate are their reports? *The European respiratory journal* 2001: 18(3): 474-481.

5. Stulbarg MS, Carrieri-Kohlman V, Gormley JM, Tsang A, Paul S. Accuracy of recall of dyspnea after exercise training sessions. *Journal of cardiopulmonary rehabilitation* 1999: 19(4): 242-248.

6. Bolton CE, Bevan-Smith EF, Blakey JD, Crowe P, Elkin SL, Garrod R, Greening NJ, Heslop K, Hull JH, Man WD-C, Morgan MD, Proud D, Roberts CM, Sewell L, Singh SJ, Walker PP, Walmsley S, British Thoracic Society Pulmonary Rehabilitation Guideline Development Group obotBTSSoCC. British Thoracic Society guideline on pulmonary rehabilitation in adults: accredited by NICE. *Thorax* 2013: 68(Suppl 2): ii1-ii30.

7. Puente-Maestu L, Palange P, Casaburi R, Laveneziana P, Maltais F, Neder JA, O'Donnell DE, Onorati P, Porszasz J, Rabinovich R, Rossiter HB, Singh S, Troosters T, Ward S. Use of exercise testing in the evaluation of interventional efficacy: an official ERS statement. *European Respiratory Journal* 2016: 47(2): 429-460.

8. ATS/ACCP Statement on cardiopulmonary exercise testing. *American journal of respiratory and critical care medicine* 2003: 167(2): 211-277.

9. Brudin L, Jorfeldt L, Pahlm O. Comparison of two commonly used reference materials for exercise bicycle tests with a Swedish clinical database of patients with normal outcome. *Clinical physiology and functional imaging* 2014: 34(4): 297-307.

10. Borg GA. Psychophysical bases of perceived exertion. *Medicine and science in sports and exercise* 1982: 14(5): 377-381.

11. Hareendran A, Leidy NK, Monz BU, Winnette R, Becker K, Mahler DA. Proposing a standardized method for evaluating patient report of the intensity of dyspnea during exercise testing in COPD. *International journal of chronic obstructive pulmonary disease* 2012: 7: 345-355.

12. Bestall JC, Paul EA, Garrod R, Garnham R, Jones PW, Wedzicha JA. Usefulness of the Medical Research Council (MRC) dyspnoea scale as a measure of disability in patients with chronic obstructive pulmonary disease. *Thorax* 1999: 54(7): 581-586.

13. Williams M, Garrard A, Cafarella P, Petkov J, Frith P. Quality of recalled dyspnoea is different from exercise-induced dyspnoea: an experimental study. *The Australian journal of physiotherapy* 2009: 55(3): 177-183.

14. Banzett RB, O'Donnell CR, Guilfoyle TE, Parshall MB, Schwartzstein RM, Meek PM, Gracely RH, Lansing RW. Multidimensional Dyspnea Profile: an instrument for clinical and laboratory research. *European Respiratory Journal* 2015: 45(6): 1681-1691.

15. Kroenke K, Spitzer RL, Williams JB. The PHQ-15: validity of a new measure for evaluating the severity of somatic symptoms. *Psychosomatic medicine* 2002: 64(2): 258-266.

**Appendix 1. Studieinformation och medgivande**

**Andfåddhet under ansträngning**

VILL DU DELTA I EN STUDIE OM MÄTNING AV ANDFÅDDHET?

**Varför tillfrågas du?**

Du har blivit kontaktad eftersom du har remitterats för att genomföra ett arbetsprov.

**Vad är studiens bakgrund och mål?**

Andfåddhet vid ansträngning är mycket vanligt och kan påverka på personens dagliga aktiviteter och livskvalitet.

Målet med denna studie är att undersöka upplevelsen och minnet av andfåddhet i samband med ett arbetsprov. Syftet är att undersöka vilka faktorer som påverkar andfåddheten och hur man skulle kunna minska graden av andfåddhet. Vi ämnar också studera sambandet mellan graden av andfåddhet och händelser såsom sjukdom och sjukhusinläggningar.

**Vad innebär studien?**

Studien består av en enkät att fylla i innan arbetsprovet. Enkäten inkluderar vilka sjukdomar och behandlingar du har och dina symtom (inklusive andfåddhet). Enkäten tar cirka 5-10 minuter att fylla i. Arbetsprovet genomförs därefter enligt klinisk rutin, med det tillägget att du får gradera svårighetsgraden av andfåddhet varannan minut under testet. I slutet av arbetsprovet kan det genomföras en nedvarvningsperiod på några minuter. Cirka en halvtimme efter arbetsprovet ber vi dig att fylla i en enkät om arbetsprovet som tar cirka 15 minuter att fylla i. En del uppgifter hämtas från din journal vid den aktuella kliniken och Patientregistret och Dödsorsaksregistretupp till 10 år framåt i tiden, gällande uppgifter om sjukdom(ar), behandling(ar), lung- och hjärtfunktion, överlevnad och sjuhusinläggningar.

**Finns det några obehag, risker och skyldigheter?**

Du utsätts inte för några risker eller andra obehag genom deltagande i studien. Ditt val påverkar inte den vård som du får eller kvaliteten på arbetsprovet. Deltagande i studien är helt frivilligt. Du kan när som helst avbryta studien och ytterligare uppgifter kommer då inte att samlas in.

**Sekretess och behandling av personuppgifter**

Den information som samlas in under studien hanteras enligt gällande sekretessbestämmelser så att obehöriga inte kan ta del av uppgifterna. Uppgifterna kodas genom att personnumret ersatts av ett studie-ID. Kodlista mellan personnummer och studie-ID förvaras inlåst i ett arkiv på kliniken. Studieresultaten kommer att presenteras anonymt så att man inte kan identifiera vilka personer som deltog i studien. Resultaten kommer att presenteras på konferenser och i allmänna och vetenskapliga tidskrifter.

**Ansvariga**

För dina personuppgifter ansvarar Landstinget Blekinge. Uppgifterna kan kombineras med uppgifter i din journal samt i offentliga svenska register för forskning, till exempel gällande sjukvård och läkemedel. I enlighet med personuppgiftslagen (PuL § 26) har du rätt att ansöka om information från hur personuppgifterna hanteras och att få eventuella felaktiga personuppgifter rättade. Detta gör du genom att kontakta personuppgiftsombudet (se nedan). Denna ansökan måste vara egenhändigt undertecknad.

**Andfåddhet under ansträngning**

Härmed intygar jag att jag läst och är införstådd med informationen ovan och att jag ger mitt medgivande att vara med i den beskrivna studien:

____________________ ____________________

Datum Underskrift

____________________ ____________________

Personnummer Namnförtydligande

Ansvarig personal som gett studieinformationen:

____________________

Datum

____________________

Underskrift

____________________

Namnförtydligande

**En kopia av båda papperna insamlas och en kopia behålls av studiedeltagaren.**

**Ansvarig och kontaktperson för studien:**

Forskningshuvudman är Landstinget Blekinge.

Kontaktperson är Magnus Ekström, läkare, medicinkliniken, Blekingesjukhuset, 371 85 Karlskrona.

Telefon: växel 0455-731000; Mail: [pmekstrom@gmail.com](mailto:pmekstrom@gmail.com)

**Frågor om personuppgifter (personuppgiftsombud):**

Helena Almtjärn, Landstinget Blekinge.

Telefon: 0455-734087; Mail: [helena.almtjarn@ltblekinge.se](mailto:helena.almtjarn@ltblekinge.se)

**Appendix 2. STUDY QUESTIONNAIRES**

**The Multidimensional Dyspnea Profile (MDP)** ©2011 R.B. Banzett

*Text att läsa första gången:*

Syftet med det här frågeformuläret är att hjälpa oss förstå hur du upplever din andning.

Det finns inga svar som är rätt eller fel. Vi är intresserade av vad du kan berätta om din egen andning.

På den här sidan ber vi dig att berätta hur obehaglig din andning känns. På en senare sida kommer vi att fråga hur intensiva eller starka dina andningsupplevelser är. Skillnaden mellan de här två aspekterna i dina andningsupplevelser kan bli tydligare om du tänker dig att du hör ett ljud, till exempel från en radio. När ljudvolymen ökar kan jag fråga hur starkt det låter eller hur obehagligt det är att höra ljudet. Till exempel kan musik som du hatar vara obehaglig även vid en låg volym och bli ännu obehagligare när volymen höjs; musik som du tycker om blir inte obehaglig ens när volymen höjs.

Skala A1

Använd den här skalan för att gradera hur **obehagliga eller besvärliga** dina andningsupplevelser är [var], hur **svår** din andning känns [kändes].

Fokusera på perioden när___________________

| ← ← | 0 | | 1 | | | 2 | | 3 | | 4 | | 5 | | 6 | | 7 | | 8 | | 9 | | 10 |  |
| --- | --- | --- | --- | --- | --- | --- | --- | --- | --- | --- | --- | --- | --- | --- | --- | --- | --- | --- | --- | --- | --- | --- | --- |
| BEHAGLIG | | NEUTRAL | |  |  | |  | |  | |  | |  | |  | |  | |  | | OLIDLIG | | |

SQ (Sensorisk kvalitet) val

Nedan följer meningar eller beskrivningar som delats in i meningar med liknande betydelse.

**Steg 1:** Sätt ett kryss för varje mening som innehåller ord som beskriver hur din andning känns [kändes] under __________________ (ange fokusperioden).

**Steg 2:** Sätt ett kryss även för *den* mening som bäst beskriver hur din andning känns [kändes].

|  | **Steg 1** | | **Steg 2** |
| --- | --- | --- | --- |
| Om *NÅGON* av beskrivningarna i meningen stämmer in på dig, välj den meningen. | STÄMMER INTE | STÄMMER | BESKRIVER BÄST |
| Min andning kräver muskelarbete ***eller*** ansträngning. |  |  |  |
| Jag får inte tillräckligt med luft ***eller*** jag kvävs ***eller*** jag vill ha mer luft. |  |  |  |
| Min bröstkorg och mina lungor känns trånga ***eller*** sammantryckta. |  |  |  |
| Min andning kräver mental ansträngning ***eller*** koncentration. |  |  |  |
| Jag andas mycket. |  |  |  |

SQ (Sensorisk kvalitet) skalor

Använd följande skalor för att gradera intensiteten på de andningsupplevelser som du har [hade]. Jämför med volymen på ett ljud som oavsett om det är behagligt eller ett obehagligt kan vara en stark upplevelse utan att vara obehagligt.

Fokusera på perioden när _____________________

| Om NÅGOT av orden i meningen stämmer in på dig ska du gradera meningen. | **INTE ALLS** | |  |  |  |  |  |  |  | DEN STÖRSTA INTENSITET SOM JAG KAN FÖRESTÄLLA MIG | | | |
| --- | --- | --- | --- | --- | --- | --- | --- | --- | --- | --- | --- | --- | --- |
| Min andning kräver muskelarbete ***eller*** ansträngning. | 0 | 1 | 2 | 3 | 4 | 5 | 6 | 7 | 8 | | 9 | 10 |  |
| Jag får inte tillräckligt med luft  ***eller*** jag kvävs  ***eller*** jag vill ha mer luft. | 0 | 1 | 2 | 3 | 4 | 5 | 6 | 7 | 8 | | 9 | 10 |  |
| Min bröstkorg och mina lungor känns  trånga ***eller*** sammantryckta. | 0 | 1 | 2 | 3 | 4 | 5 | 6 | 7 | 8 | | 9 | 10 |  |
| Min andning kräver  mental ansträngning ***eller*** koncentration. | 0 | 1 | 2 | 3 | 4 | 5 | 6 | 7 | 8 | | 9 | 10 |  |
| Jag andas mycket. | 0 | 1 | 2 | 3 | 4 | 5 | 6 | 7 | 8 | | 9 | 10 |  |
| Annat* | 0 | 1 | 2 | 3 | 4 | 5 | 6 | 7 | 8 | | 9 | 10 |  |

*Om det behövs kan du lägga till ytterligare beskrivningar av dina andningsupplevelser.

A2-skalor

När din andning inte känns som den brukar kan det hända att du upplever vissa ”känslor”. Använd skalorna nedan till att förklara hur dina andningsupplevelser har fått dig att känna dig – sätt noll på de känslor du inte har känt.

Fokusera på dina känslor under perioden när _____________________

|  | **INTE ALLS** | |  |  |  |  |  |  |  | **MESTA TÄNKBARA** | | |
| --- | --- | --- | --- | --- | --- | --- | --- | --- | --- | --- | --- | --- |
| Deprimerad | 0 | 1 | 2 | 3 | 4 | 5 | 6 | 7 | 8 | 9 | 10 |  |
| Ängslig | 0 | 1 | 2 | 3 | 4 | 5 | 6 | 7 | 8 | 9 | 10 |  |
| Frustrerad | 0 | 1 | 2 | 3 | 4 | 5 | 6 | 7 | 8 | 9 | 10 |  |
| Arg | 0 | 1 | 2 | 3 | 4 | 5 | 6 | 7 | 8 | 9 | 10 |  |
| Rädd | 0 | 1 | 2 | 3 | 4 | 5 | 6 | 7 | 8 | 9 | 10 |  |
| Annat? | 0 | 1 | 2 | 3 | 4 | 5 | 6 | 7 | 8 | 9 | 10 |  |

**mMRC breathlessness scale**

0 = Jag blir andfådd när jag anstränger mig rejält, inte när jag tar en snabb promenad eller går i uppförsbacke.

1 = Jag blir andfådd när jag tar en snabb promenad eller går i en uppförsbacke.

2 = Jag blir andfådd när jag går på slät mark i samma takt som någon i min ålder. 3 = Jag blir så andfådd när jag går på slät mark att jag måste stanna upp trots att jag själv bestämmer takten.

4 = Jag blir andfådd när jag tvättar eller klär mig.
