# Supplementary figures and images for: Effect of the trajectory of exertional breathlessness on symptom recall and anticipation: A randomized controlled trial
Source: PLoS One. 2020 Sep 11;15(9):e0238937. doi: 10.1371/journal.pone.0238937 (PMC7486077; doi:10.1371/journal.pone.0238937)

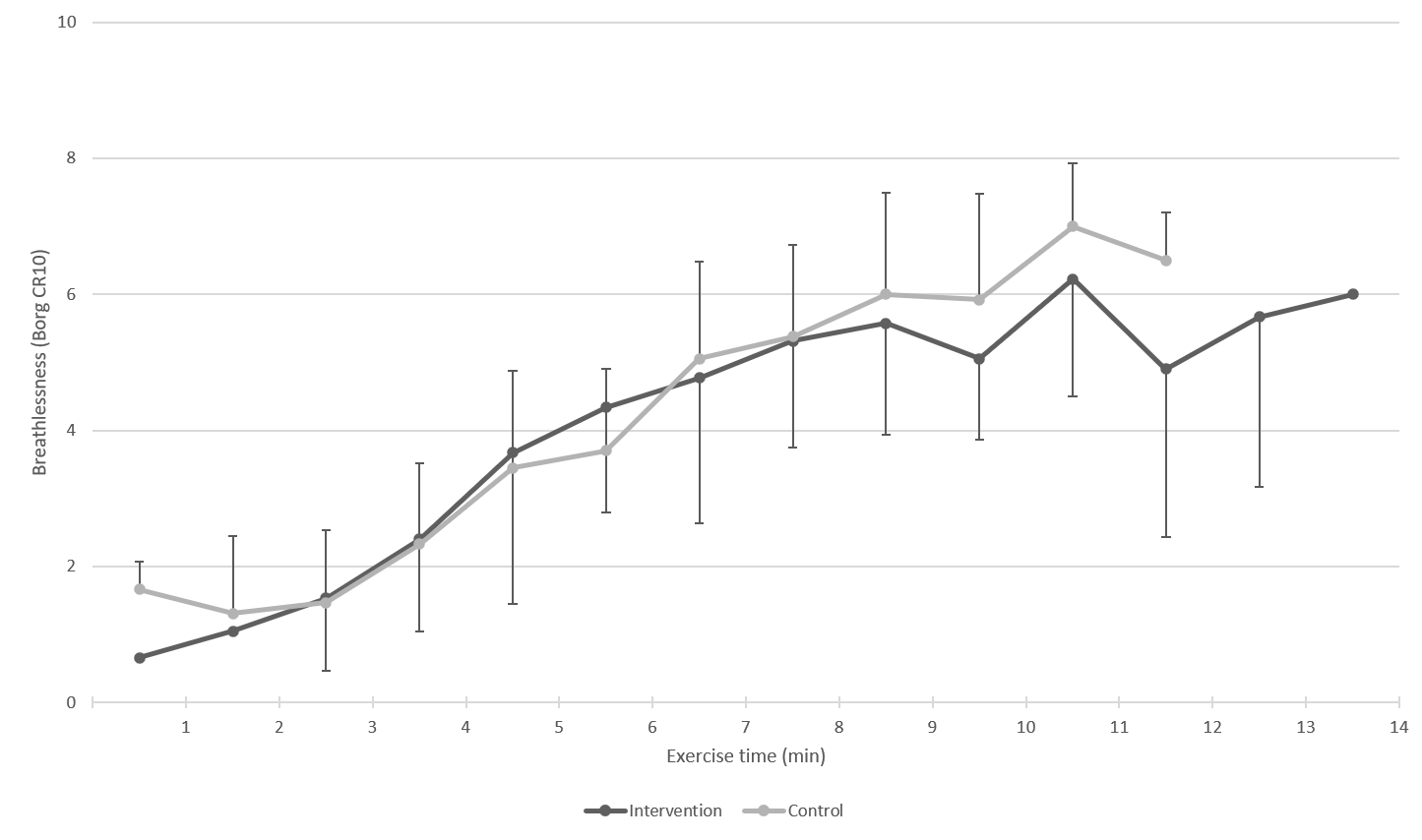

Supplement: S1 Fig — Mean trajectory of breathlessness by study group. Each point shows the mean breathlessness score per minute (Borg CR10). Peak breathlessness was similar between the groups. End breathlessness was significantly lower in the intervention group compared to the control group, mean difference 0.96 (95% CI, 0.24 to 1.67). Error bars shows 1 SD. The first two SD for the intervention group was omitted from the graph for legibility (0.66 for 0.5 minutes and 1.05 for 1.5 minutes respectively). Abbreviations: SD, standard deviation; Borg CR10, Borg Category-ratio scale (0–10). (TIF) [file pone.0238937.s003.tif]
