# Supplementary material for: Effect of the trajectory of exertional breathlessness on symptom recall and anticipation: A randomized controlled trial
Source: PLoS One. 2020 Sep 11;15(9):e0238937. doi: 10.1371/journal.pone.0238937 (PMC7486077; doi:10.1371/journal.pone.0238937)
Supplement: Attachment — Submitted filename: Response to reviewers.docx [file pone.0238937.s004.docx]

**Revision. Authors response to reviewers, point-by-point**

**Elmberg et al.** **“Effect of the trajectory of exertional breathlessness on symptom recall and anticipation: a randomized controlled trial”**

**Reviewer #1:**

The study protocols and aims are also very explicit, and the null finding discussed in a thorough manner.

Thank you.

1. Patient population not described in abstract nor methods – this makes the reader curious as to why they were referred for exercise testing? There must have been other existing conditions that lead to this prescription for an exercise test? This is somewhat covered in the table of patient demographics, so it would be useful to have an in-text reference to this. A short overview in the methods section text would also be helpful.

Thank you for this suggestion. We agree and hade added the following line to the methods section, page 4, paragraph 3:

*“The reason for referral were in most cases suspected chronic coronary syndrome. Other reasons for the test were suspected exercise induced arrhythmias, occupational health screenings and breathlessness.”*

2. The question “How are your physical capacity” does not make grammatical sense in English – is there a more appropriate translation (I imagine this question was delivered in Swedish originally)?

Yes, it was translated from Swedish. We agree and have changed this to, page 7, paragraph 2:

“*Estimate your physical capacity”*

3. Statistical analyses: Were non-normally distributed variables compared using non-parametric tests? It is stated that they are described as a mean ± IQR, but were they also compared between groups in a non-parametric fashion instead of t-tests? It is not clear from the current description in the methods. It would also be helpful to have an additional column in the table of results that depicts which test (with the parametric or non-parametric nature) was employed for each variable.

Thank you, we have compared durations (that were expected to be non-normally distributed) with Wilcoxon rank-sum test instead (table 2). Durations in table 2 were Wilcoxon rank-sum test were used were marked with “*”. We also confirmed normal distribution, of the variables were t-tests were used, with histogram plots. A line was added to reflect this, page 8, paragraph 1:

*“Normal distribution was confirmed using histogram plots. Non-normal continuous variables were compared using Wilcoxon Rank-Sum test.”*

4. Table 4 is a little confusing. Could this be adjusted to two lines, as I understand the last two lines are part of the same regression analysis, with the column on the left depicting whether the estimates were calculated using separate or simultaneous regressions?

Thank you for this comment. We understand how this could cause confusion and have fused the last two rows in table 4, page 14.

5. Figure 2 would benefit from error bars on the graph, so that it is possible to visualise the variability in participant responses at each time point. A vertical line at the end of the incremental exercise might also be helpful to see when incremental exercise was terminated. Alternatively (or additionally in a second Figure panel), it could be helpful to see breathlessness scores plotted against exercise intensity - possibly as a percentage of maximum for consistency across participants.

We thank the reviewer for raising these points. Error bars have been added. Importantly, the same exercise protocols were used and the increases in exercise intensity per time unit were similar in both group due to the randomization. Therefore, plotting by exercise intensity such as W or W%max) on the x-axis give similar results and, in our opinion, does not provide anything beyond the data presented in the current graph. The termination time points (and work rates) varied between individuals in both groups and, therefore, it is not feasible to plot these. The figure provides the actual values for different time points throughout the exercise tests for both randomized groups.

6. How were the missing values dealt with? Participants excluded, a correction method used? It would be helpful if this was described.

Due to the standardized test and assessment protocol, there were very few missing data points (as described in Methods page 10). We agree this needs clarification and have added a statement that no data were imputed, on page 12, paragraph 3. The number of missing data points has also been corrected.

7. It might also be useful to plot and test the correlation between peak and observed breathlessness, to see if this is the reason for no additional variance being explained when both regressors are included in the analysis together.

We think that the reviewer here asks about the correlation between the peak and end breathlessness; that correlation, using linear regression, was 0.62 (please see the scatterplot below, r = 0.68). The correlation was quite high so we agree with the reviewer that this probably at least partly explains the lack of added variance explained. There is a short comment on ‘end breathlessness’ not being independently associated (when adjusting for the peak value) with recall in the discussion section, page 15, paragraph 3

**Reviewer #2:**

Abstract
No comments. Reads clearly enough.

Thank you.

Intro
Premise of the study well developed.

Thank you.

Methods

1. Authors should state why these patients were referred for CPX. Examination of Table shows remarkable absence of morbidity in study population…most were healthy?

Thank you, we agree and have added a line on this as also suggested by Reviewer 1, page 4, paragraph 3. The participants were reasonably healthy. Most tests were done because of suspected chronic coronary syndrome. However, participants with a higher pre test probability (meaning more comorbidities) of disease often did other types of tests like myocardial perfusion imaging.

2. Authors collected much data that was not reported. One item in particular seemed strange (ll. 124-5: how would subjects know a priori what maximal exercise test would feel like?)

We anticipated that participants would be able to estimate how it would feel as most people in Sweden are used to biking. As we did not use this data we have deleted it from the manuscript.

3. I missed results of questions posed in ll. 148-9. What would their relevance be?

Results are reported in table 3. It was hypothesized that participants who were randomized to the intervention group would anticipate a lower expected (future) breathlessness/exertion (the rationale being that they would recall being less breathless and therefore might have predicted that they would become less breathless in a repeated test) and having a higher physical capacity if a new test were to be done.

4. Frequency of dyspnea & perceived exertion assessments in text (up to lines 140) does not match results shown in Table. Text does not state these symptoms rated during recovery, yet such data appears in Table 2 even for control group (unless this is what authors meant whent they wrote “more often when needed” in line 139?).

Thank you, we have corrected this. Breathlessness, and chest pain as needed, was also inquired about during recovery. Exertion was however not. We have corrected table 2 and added the following line, page 6, paragraph 3:

*“breathlessness intensity was self-reported by the participant before the test, every 2 minutes during exercise, at peak exercise, at the end of the intervention period, and at the completion of the test on a Borg CR10 scale”*

5. Post-exercise question posed in ll. 153-4 were not asked pre-exercise, were they?

The reviewer is correct, only the question on expected intensity of breathlessness were asked both before and after the test.

Results
These are reported clearly, Tables complementing text, except Table 4 which only causes confusion. My only question stems from Table 2: how did they obtain Borg CR 10 rating 6.80 (1.53) in controls during recovery, & why did it differ from peak exercise 6.53? This relates to my bullet points in Methods above.

Thank you for noticing, this is mostly likely caused by some measurements being taken immediately after cessation of exercise, when the oxygen debt is at its highest with the patient having a further increase of ventilation. In this setting the estimated breathlessness rating can be higher than at peak exercise. The same phenomena likely could be seen in some patients in the intervention group also. We have added a line in the discussion section regarding this page 16, paragraph 1:

*“Peak breathlessness was marginally (non-significantly) higher immediately post exercise in the control group, which was likely caused by the buildup of oxygen debt with the participants likely having further increased ventilation after cessation of exercise.”*

Discussion
In reality, this could be shortened considerably, particularly paragraph on implications. It is here that authors introduce concept of “larger total amount of breathlessness” as if they’re reporting area under curve. In reality, they had intervention group exercise 2 more minutes to cool down & obtained another dyspnea rating, which they did not to in controls (ll. 168-70). Authors state the obvious in ll. 294-5, as this result was inherent in study design.

Thank you, we have struck a few lines from the discussion section that we felt were superfluous. We have also changed “larger amount of breathlessness” to “longer duration of breathlessness”.

Minor Points:
1. Line 67: expected or anticipated is better word than “predicted”.

Thank you, we agree and have changed this on page 3, paragraph 3 and throughout the manuscript.

2. NOTE that Figure 2 is not mentioned in text of manuscript; only in Figure legend.

Thank you for noticing this, we have added the following line, page 10, paragraph 1:

*“Figure 2 shows the mean trajectory of breathlessness by study group.”*

**Reviewer #3:**

The aims of the randomized controlled clinical trial were to (1) test the effect of adding two minutes of low intensity exercise (intervention) on breathlessness, (2) analyze the impact of peak and end exertional breathlessness intensity on breathlessness recall. No significant difference was observed in recalled breathlessness intensity between the arms. However, the intervention arm had significantly lower end breathlessness. The manuscript was written with clarity and a high level of detail.

Minor revisions:
1- Line 162: State the underlying covariance structure used in the mixed effects linear model and the criteria for selecting it.

This was a typo. Mixed effect linear models were used in an earlier draft to create figure 2. We have removed this line as we now use the actual values instead.

2- Line 177: Indicate the statistical testing method which achieves 80% power and if the alpha level was one- or two-sided.

This has been clarified as suggested, by adding “using Student’s t-test” and specifying that the p-values were two-sided.

3- Provide more precise p-values than “p < 0.05.”

We have added precise p-values, page 10, paragraph 3.

4- Line 229: Typographical error sensitivity analysis results are not located in Table 1.

Thank you, we have corrected this.
