# Supplementary material for: Effect of the trajectory of exertional breathlessness on symptom recall and anticipation: A randomized controlled trial
Source: PLoS One. 2020 Sep 11;15(9):e0238937. doi: 10.1371/journal.pone.0238937 (PMC7486077; doi:10.1371/journal.pone.0238937)
Supplement: Attachment — Submitted filename: Response to reviewers.docx [file pone.0238937.s005.docx]

**Revision. Authors response to editor, point-by-point**

**Elmberg et al.** **“Effect of the trajectory of exertional breathlessness on symptom recall and prediction: a randomized controlled trial”**

**Academic editor**

1. Data availability: PLOS guidelines stipulate that a ‘minimal dataset’ must be shared with a publication (guidelines: <https://journals.plos.org/plosone/s/data-availability>). This minimal dataset includes the key data values that were inferred upon in the manuscript, such as the underlying values that went into the group mean and regression values reported in Tables 2-4. Ideally data would be placed in a public access repository (strongly recommended), or alternatively the data can be shared within the supplementary material. It is not necessary for authors to submit their entire dataset, for instance any personal or identifying information should not be submitted, and as the sensitive data (such as health information) was used in a purely descriptive sense in this manuscript, there is no need for this data to be made available. Therefore, it should be possible to provide spreadsheets of the exercise data with no subject identification whatsoever, such that participants could never be identified. If the authors can provide reference to documentation from the relevant ethics committee that states making the data publicly available in this form is still not possible, only then would it be considered appropriate for interested parties to initiate contact to request access to the data. However, providing the details of the corresponding author for this purpose is not considered appropriate in the data sharing policies provided by PLOS. Instead it is required that the authors provide contact information for a data access committee, ethics committee, or other institutional body to which data requests may be sent (<https://journals.plos.org/plosone/s/data-availability>). This ensures a more stable data access point than a single researcher.

Thank you. We have anonymized our data and published a minimal dataset at Hardvard dataverse. The dataset contains all necessary data to replicate the study findings. The data availability statement has been updated to reflect this:

“All relevant data are available at the Hardvard dataverse repository at <https://doi.org/10.7910/DVN/QMQOCV>”

2. Figure 2: Regarding a previous point raised by the reviewers, plotting the values as a function of time does not represent the data protocol used in this study. It would be better if the data were plotted as a value that could be standardised across participants, such as a percentage of maximal effort, such that it can be clearly delineated which breathlessness values were from the incremental exercise and which were from the intervention period following the exercise. With individuals stopping at different points in time, plotting breathlessness as a function of time is not informative. Additionally, it would be more helpful to the reader if all error bars were plotted rather than having some omitted as it is currently formatted.

Thank you we have edited Figure 2 to now show percentage of maximal Watt on the X axis. In retrospect we believe that this much improves the figure and thank the editor for this valuable suggestion. We have also added error bars for all data points. As we nonetheless believe that it could be of some interest to see the breathlessness score relative to the actual time we have included “the old” Figure 2 under supplements as Figure 3.

3. This is still very confusing for a reader to interpret, as stated in the previous reviewer comments. Perhaps changing the ‘Adjusted for’ column to ‘Regression model’, and describing the rows as either a single regressor or using separate models (row 1), and using multiple regressors or one model (row 2) or something similar might provide more clarity here?

We agree that table 4 required further clarification and have adjusted it as below. We hope that it will now be more understandable.

|  | **Association with recalled breathlessness, linear regression Beta-coefficient (95% CI) and r^2^** | |
| --- | --- | --- |
| **Regression model:** | **Peak** | **Last** |
| Each separate | 0.68 (0.44-0.92)  r^2^ = 0.43 | 0.41 (0.18-0.64)  r^2^ = 0.22 |
| Both (multivariable) | 0.75 (0.36-1-14)  r^2^ = 0.43 | -0.07 (-0.39-0.25)  r^2^ = 0.43 |

4. It is not currently clear how many of the points within the discussion paragraph on the study implications (beginning on line 337) have direct relevance to the results presented in this paper. In particular, nowhere in the results is the relationship between recalled and experienced breathlessness shown, nor that peak breathlessness is closely associated with future anticipated breathlessness. This paragraph could be removed from the manuscript, as discussed in the previous round of reviewer comments.

Thank you. We have reworked some phrases and considerably shortened this paragraph to be more relevant to the study findings. Regarding “experienced breathlessness” it was meant to illustrate that the intervention group, who biked longer compared to the control group, did not have a higher recalled breathlessness. We however agree that this could be confusing, especially considering the study hypothesis and different phrasing and have thus deleted this.

Results regarding “anticipated future breathlessness” is reported on page 14, paragraph 1:

“Future anticipated breathlessness if a new stress test were to be performed were also more strongly related to peak than end breathlessness although the associations were weaker (r2 = 0.16 vs. 0.11; p = 0.006 and 0.025 respectively). Again, end breathlessness did not increase the variance of recalled breathlessness explained above that for peak breathlessness.“

We hope that this is satisfactory as we believe that the findings could potentially have implications on cardiopulmonary exercise programs and that this should be discussed to at least some extent.
